# The Gaucher Earlier Diagnosis Consensus point-scoring system for children and young adults: a retrospective and prospective evaluation in Korea

**DOI:** 10.1186/s13023-025-03891-1

**Published:** 2025-11-10

**Authors:** Seung Min Hahn, Minseok Kim, Youna Kim, Jiyoung Oh, Se Hee Kim, Hong Koh, Sung-Eun Kim, Nack-Gyun Chung, Ye Jee Shim, Hawk Kim, Jae Min Lee, Sung-Soo Yoon, Ho Joon Im, Hyoung Jin Kang, Young Rok Do, Chung Mo Nam, Chuhl Joo Lyu

**Affiliations:** 1 Department of Pediatrics, Yonsei University College of Medicine, Seoul, Republic of Korea; 2 Department of Pediatric Hematology-Oncology, Yonsei Cancer Center, Severance Hospital, Yonsei University Health System, 50-1 Yonseiro, Seodaemun-gu, Seoul, 03722 Republic of Korea; 3 Division of Biostatistics, Department of Biomedical Systems Informatics, Yonsei University College of Medicine, Seoul, Republic of Korea; 4 Academic Research Organization of Clinical Trials Center, Severance Hospital, Yonsei University Health System, Seoul, Republic of Korea; 5 Pediatric Neurology, Department of Pediatrics, Epilepsy Research Institute, Severance Children’s Hospital, Yonsei University College of Medicine, Seoul, Republic of Korea; 6 Department of Pediatrics, School of Medicine, Kyungpook National University, Daegu, Republic of Korea; 7 Department of Pediatrics, Catholic Hematology Hospital, Seoul St. Mary’s Hospital, College of Medicine, The Catholic University of Korea, Seoul, Republic of Korea; 8 Division of Hematology, Gachon University Gil Medical Center, Gachon University College of Medicine, Incheon, Republic of Korea; 9 Department of Pediatrics, Pusan National University School of Medicine, Pusan National University Children’s Hospital, Yangsan-si, Republic of Korea; 10 Division of Hematology/Medical Oncology, Department of Internal Medicine, Department of Tumor Biology, Cancer Research Institute, Seoul National University Hospital, Seoul National University College of Medicine, Seoul, Republic of Korea; 11 Division of Pediatric Hematology/Oncology, Department of Pediatrics, Asan Medical Center Children’s Hospital, University of Ulsan College of Medicine, Seoul, Republic of Korea; 12 Department of Pediatrics, Seoul National University College of Medicine, Seoul National University Cancer Research Institute, Seoul National University Children’s Hospital, Seoul, Republic of Korea; 13 Division of Hemato-Oncology, Department of Medicine, Dongsan Medical Center, Keimyung University, Daegu, Republic of Korea; 14 Department of Preventive Medicine, Yonsei University College of Medicine, Seoul, Republic of Korea

**Keywords:** Gaucher disease, Lysosomal storage disorder, Early diagnosis

## Abstract

**Purpose:**

Gaucher disease (GD) is an autosomal recessive condition caused by insufficient glucocerebrosidase activity. The Gaucher Earlier Diagnosis Consensus (GED-C) initiative created a point-scoring system (PSS) to facilitate the early identification of GD based on significant indicators and covariables. This study aimed to evaluate the applicability and utility of the GED-C PSS in pediatric and young adult patients in Korea.

**Results:**

This study included both retrospective analysis and prospective recruitment. Subject recruitment involved 14 sites across 13 hospitals in Korea, where patients of any age meeting GED-C criteria were recruited, and blood samples were collected. Data of 513 subjects were analyzed and two patients were confirmed to have GD during prospective enrollment. The median age of participants was 10 years (range: 1 month to 40 years). Receiver operating characteristic analysis revealed a cutoff point of 6.5 for GED-C PSS (area under the curve of 0.9883) demonstrated high sensitivity (1.0) and specificity (0.97). A histogram indicated that the PSS scores of the two confirmed GD patients were distinct from those of other participants.

**Conclusions:**

The study suggests that GED-C PSS shows potential for the early diagnosis of GD, supporting its broader clinical use for both children and adults.

## Background

Gaucher disease (GD) is an autosomal recessive metabolic disorder that impairs glycolipid recycling in cells [1, 2] due to deficient activity of the lysosomal enzyme glucocerebrosidase, leading to the accumulation of glucosylceramide in macrophages [1–3] known as Gaucher cells. This metabolic impairment causes cellular dysfunction and clinical abnormalities, primarily affecting the bone marrow, spleen, and liver, though other organs can also be involved. The enzyme deficiency may also impact cells beyond macrophages, such as hematopoietic progenitor cells, erythrocytes, mesenchymal cells, and hepatocytes, contributing to the diverse presentations of GD [2].

Over 400 variants of the pathogenic *GBA1* gene located on chromosome 1 (1q21) have been identified as causes of GD [4, 5]. The phenotype of GD is variable; based on the extent and age of neurological involvement, three phenotypes have been suggested to classify GD: type 1 (nonneuronopathic), type 2 (acute neuronopathic), and type 3 (chronic neuronopathic). Type 1 is the most prevalent form of GD, typically presenting without neurologic impairment but with various manifestations such as hepatosplenomegaly, cytopenia, bone pain, or pulmonary involvement. Type 2 GD is the rarest form, known as the acute neuropathic type, and typically presents in infancy. Type 3 GD is characterized by a more indolent progression of neurologic impairment [1, 2].

Definitive diagnosis of GD can be confirmed by observing decreased glucocerebrosidase enzyme activity in the presence of a biallelic pathogenic *GBA* variants. While newborn screening (NBS) programs in various countries have demonstrated the feasibility and benefits of early GD detection and intervention [6–10], NBS for GD may lead to identifying individuals who do not require immediate treatment due to the disease’s variable presentation, as noted by the Delphi expert panel [11].

The Gaucher Earlier Diagnosis Consensus (GED-C) initiative suggested major signs and covariables of relevance in early GD to facilitate diagnosis by Delphi methodology [12]. For type 1 GD, seven major signs (splenomegaly, thrombocytopenia, bone manifestations, anemia, hyperferritinemia, hepatomegaly, and gammopathy) and two major covariables (family history, Ashkenazi-Jewish ancestry) were identified. For type 3 GD, nine major signs (splenomegaly, oculomotor disturbances, thrombocytopenia, epilepsy, anemia, hepatomegaly, bone pain, motor disturbances, and kyphosis) and one major covariable (family history) were identified. These parameters may help non-specialists identify GD and increase their level of suspicion [2, 12]. The proposed prototype point scoring system (PSS) by the GED-C panel effectively distinguished GD patients from those with overlapping symptoms [13–15].

According to rare disease registry data from the Korea Disease Control and Prevention Agency, annual newly diagnosed GD patients were only 1 in 2020, and 4 in 2021. Efforts to implement neonatal screening for lysosomal storage diseases, including GD, have only recently begun, and the disease still appears to be underdiagnosed in South Korea. This study aimed to determine the optimal cutoff value for GED-C PSS based on symptoms and test findings for early GD diagnosis in Korean patients. Additionally, we sought to identify the clinical manifestations of GD in Koreans and assess the utility of GED-C PSS, particularly in pediatric, adolescent, and young adult populations, given the limited research on these groups.

## Methods

### Point scoring

The GED-C PSS was used to estimate scores for each participant suspected of having GD (Table 1). Score for each factor was stratified (3 points, 2 points, 1 point, and 0.5 points) based on the likelihood of association with GD as determined by GED-C consensus. There is currently no validated threshold for the GED-C PSS score to be used as a screening cutoff. To broaden the range of patients eligible for screening, our research team planned to include all individuals with a GED-C PSS score of 2 points or higher.

**Table 1:** Gaucher Earlier Diagnosis Consensus (GED-C) regarding signs and co-variables with their scores in point-scoring system (PSS). If the total PSS corresponding is 2 or higher, the individual is eligible to enroll in the prospective or retrospective study

Score	Sign or co-variable
Major signs and co-variables	
3 points	Splenomegaly (≥ 3 × normal) Disturbed oculomotor function (slow horizontal saccades with unimpaired vision)
2 points	Thrombocytopenia, mild or moderate: 50 × 10^3^/μL ≤ platelet count < 150 × 10^3^/μL Bone issues, including pain, crises, avascular necrosis and fractures Family history of Gaucher disease Anemia, mild or moderate: 1 ≤ age < 2: 8.0 g/dL ≤ hemoglobin < 10.5 g/dL 2 ≤ age < 12: 8.0 g/dL ≤ hemoglobin < 11.5 g/dL 12 ≤ age < 19: 8.0 g/dL ≤ hemoglobin < 12.0 g/dL 19 ≤ age: 8.5 g/dL ≤ hemoglobin < 12.0 g/dL) Hyperferritinemia, mild or moderate: 300 ng/mL ≤ serum ferritin < 1000 ng/mL Jewish ancestry Disturbed motor function (impairment of primary motor development) Hepatomegaly, mild or moderate (≤ 3 × normal) Myoclonus epilepsy Kyphosis Gammopathy—monoclonal or polyclonal
1 point	Anemia, severe: 1 ≤ age < 19: hemoglobin < 8.0 g/dL 19 ≤ age: hemoglobin < 8.5 g/dL Hyperferritinemia, severe: Serum ferritin ≥ 1000 ng/mL) Hepatomegaly, severe (> 3 × normal) Thrombocytopenia, severe: Platelet count < 50 × 103/μL)
Minor signs and co-variables	
0.5 points	Gallstones Bleeding, bruising or coagulopathy Leukopenia Cognitive deficit Low bone mineral density Growth retardation including low body weight Asthenia Cardiovascular calcification Dyslipidemia Elevated ACE levels Fatigue Pulmonary infiltrates Age < 19 years Family history of Parkinson’s disease Blood relative who died of fetal hydrops and/or with diagnosis of neonatal sepsis of uncertain etiology

### Patients and data collection

This study involved both retrospective data analysis and prospective patient recruitment. The data collection and patient enrollment were performed between May 2019 and November 2023. Individuals already diagnosed with GD were excluded from the study.

In the retrospective study, data from patients below 19 years of age who visited Severance Hospital within the 5 years prior to study approval month (Visit 0) were reviewed. Patients exhibiting symptoms suggestive of GD and meeting GED-C criteria (Table 1) were included. If the total PSS corresponding was 2 or higher, the individual was eligible to enroll in the study. When these patients subsequently visited the outpatient clinic (Visit 1), approximately 3 mL of venous blood was drawn using a syringe for a dry blood smear test to confirm GD. Blood was smeared on a filter card and sent to a central laboratory.

For the prospective study, 14 sites across 13 hospitals in Korea recruited patients. Since the GED-C criteria may not be familiar to all clinicians, we provided thorough education and training to the investigators at the start of the study to ensure a clear understanding of the criteria. This training was repeated as necessary to maintain consistent application of the criteria. Additionally, we implemented a process of data verification during data collection to ensure accuracy and consistency. Patients of pediatric age and young adults who visited outpatient clinics or were hospitalized and exhibited symptoms suggestive of GD and met the GED-C criteria (Table 1) were recruited and scored. Subjects with PSS 2 or higher were eligible to enroll in the study. During their visit (Visit 1), blood was drawn and tested.

### Statistical analysis

A receiver operating characteristic (ROC) curve was used to summarize diagnostic performance in terms of sensitivity and specificity of various PSS cutoff values for distinguishing between GD and non-GD cases. The area under the ROC curve (AUC) and the corresponding 95% confidence interval (CI) were calculated using the bootstrapping method. The Youden index (sensitivity + specificity—1) was used to determine the statistically derived optimal cutoff on the ROC curve for diagnosing GD [16]. All statistical analyses were performed using R (version 4.3.0).

## Results

Of the 518 registered participants, 513 were analyzed after excluding five due to dropouts and duplicates. The retrospective and prospective groups comprised 162 and 351 participants, respectively. The median age of participants was 10 years (range: 1 month to 40 years). Of the total number of patients, 478 visited for hematologic concerns, and 35 were referred from other specialties for consultation. Most common hematologic abnormalities were thrombocytopenia (55%), anemia (67%), and leukopenia (99%) (Table 2). Two patients were confirmed to have GD.

**Table 2:** Patients’ characteristics and demographics according to Gaucher early diagnosis consensus, point-scoring system

Characteristic	Score	Non-Gaucher (N = 511)	Gaucher (N = 2)
Cohort			
Prospective		349 (68%)	2 (100%)
Retrospective		162 (32%)	0 (0%)
Overall PSS score			
Mean (SD)		3.47 (1.26)	9.00 (3.54)
Range		2.00, 9.00	6.50, 11.50
Median (IQR)		3.00 (2.50, 4.50)	9.00 (7.75, 10.25)
Assessed GED-C PSS sign or co-variables Splenomegaly (≥ 3 × normal)			
Yes	3 points	17 (3.3%)	1 (50%)
No		472 (92%)	1 (50%)
Unknown		22 (4.3%)	0 (0%)
Disturbed oculomotor function (slow horizontal saccades with unimpaired vision)			
Yes	3 points	2 (0.4%)	0 (0%)
No		507 (99%)	2 (100%)
Unknown		2 (0.4%)	0 (0%)
Thrombocytopenia			
Mild or Moderate	2 points	173 (34%)	1 (50%)
Severe	1 points	108 (21%)	1 (50%)
No		225 (44%)	0 (0%)
Unknown		5 (1.0%)	0 (0%)
Anaemia			
Mild or Moderate	2 points	285 (56%)	0 (0%)
Severe	1 points	58 (11%)	0 (0%)
No		163 (32%)	2 (100%)
Unknown		5 (1.0%)	0 (0%)
Leukopenia			
Yes	0.5 points	160 (31%)	1 (50%)
No		346 (68%)	1 (50%)
Unknown		5 (1.0%)	0 (0%)
Hyperferritinaemia			
Mild or Moderate	2 points	40 (7.8%)	1 (50%)
Severe	1 points	25 (4.9%)	0 (0%)
No		207 (41%)	1 (50%)
Unknown		239 (47%)	0 (0%)
Hepatomegaly			
Mild or Moderate	2 points	59 (12%)	1 (50%)
Severe	1 points	2 (0.4%)	0 (0%)
No		426 (83%)	1 (50%)
Unknown		24 (4.7%)	0 (0%)
Dyslipidemia			
Yes	0.5 points	13 (2.5%)	0 (0%)
No		437 (86%)	1 (50%)
Unknown		61 (12%)	1 (50%)
Elevated angiotensin-converting enzyme levels			
Yes	0.5 points	2 (0.4%)	0 (0%)
No		68 (13%)	0 (0%)
Unknown		441 (86%)	2 (100%)
Family history of Gaucher disease			
Yes	2 points	0 (0%)	0 (0%)
No		505 (99%)	2 (100%)
Unknown		6 (1.2%)	0 (0%)
Disturbed motor function (impairment of primary motor development)			
Yes	2 points	1 (0.2%)	2 (100%)
No		509 (100%)	0 (0%)
Unknown		1 (0.2%)	0 (0%)
Myoclonus epilepsy			
Yes	2 points	13 (2.5%)	1 (50%)
No		496 (97%)	1 (50%)
Unknown		2 (0.4%)	0 (0%)
Cognitive deficit			
Yes	0.5 points	5 (1.0%)	1 (50%)
No		500 (98%)	0 (0%)
Unknown		6 (1.2%)	1 (50%)
Gammopathy—monoclonal or polyclonal			
Yes	2 points	3 (0.6%)	0 (0%)
No		94 (18%)	1 (50%)
Unknown		414 (81%)	1 (50%)
Bone issues, including pain, crises, avascular necrosis, and fractures			
Yes	2 points	6 (1.2%)	0 (0%)
No		492 (96%)	2 (100%)
Unknown		13 (2.5%)	0 (0%)
Kyphosis			
Yes	2 points	0 (0%)	0 (0%)
No		505 (99%)	2 (100%)
Unknown		6 (1.2%)	0 (0%)
Low bone mineral density			
Yes	0.5 points	3 (0.6%)	0 (0%)
No		77 (15%)	1 (50%)
Unknown		431 (84%)	1 (50%)
Jewish ancestry			
Yes	2 points	0 (0%)	0 (0%)
No		505 (99%)	2 (100%)
Unknown		6 (1.2%)	0 (0%)
Gallstones			
Yes	0.5 points	5 (1.0%)	0 (0%)
No		215 (42%)	2 (100%)
Unknown		291 (57%)	0 (0%)
Bleeding, bruising, or coagulopathy			
Yes	0.5 points	55 (11%)	0 (0%)
No		453 (89%)	2 (100%)
Unknown		3 (0.6%)	0 (0%)
Growth retardation including low body weight			
Yes	0.5 points	10 (2.0%)	1 (50%)
No		500 (98%)	1 (50%)
Unknown		1 (0.2%)	0 (0%)
Asthenia			
Yes	0.5 points	4 (0.8%)	0 (0%)
No		504 (99%)	2 (100%)
Unknown		3 (0.6%)	0 (0%)
Cardiovascular calcification			
Yes	0.5 points	0 (0%)	0 (0%)
No		109 (21%)	1 (50%)
Unknown		402 (79%)	1 (50%)
Fatigue			
Yes	0.5 points	17 (3.3%)	0 (0%)
No		488 (95%)	1 (50%)
Unknown		6 (1.2%)	1 (50%)
Pulmonary infiltrates			
Yes	0.5 points	0 (0%)	0 (0%)
No		179 (35%)	1 (50%)
Unknown		332 (65%)	1 (50%)
Age ≤ 18 years			
Yes	0.5 points	448 (88%)	1 (50%)
No		63 (12%)	1 (50%)
Unknown		0 (0%)	0 (0%)
Family history of Parkinson disease			
Yes	0.5 points	0 (0%)	0 (0%)
No		499 (98%)	2 (100%)
Unknown		12 (2.3%)	0 (0%)
Blood relative who died of fetal hydrops and/or with diagnosis of neonatal sepsis of uncertain etiology			
Yes	0.5 points	0 (0%)	0 (0%)
No		501 (98%)	2 (100%)
Unknown		10 (2.0%)	0 (0%)

GED-C, Gaucher Early Diagnosis Consensus; PSS, point-scoring system

### ROC analysis and PSS cutoff value

**Fig. 1:**
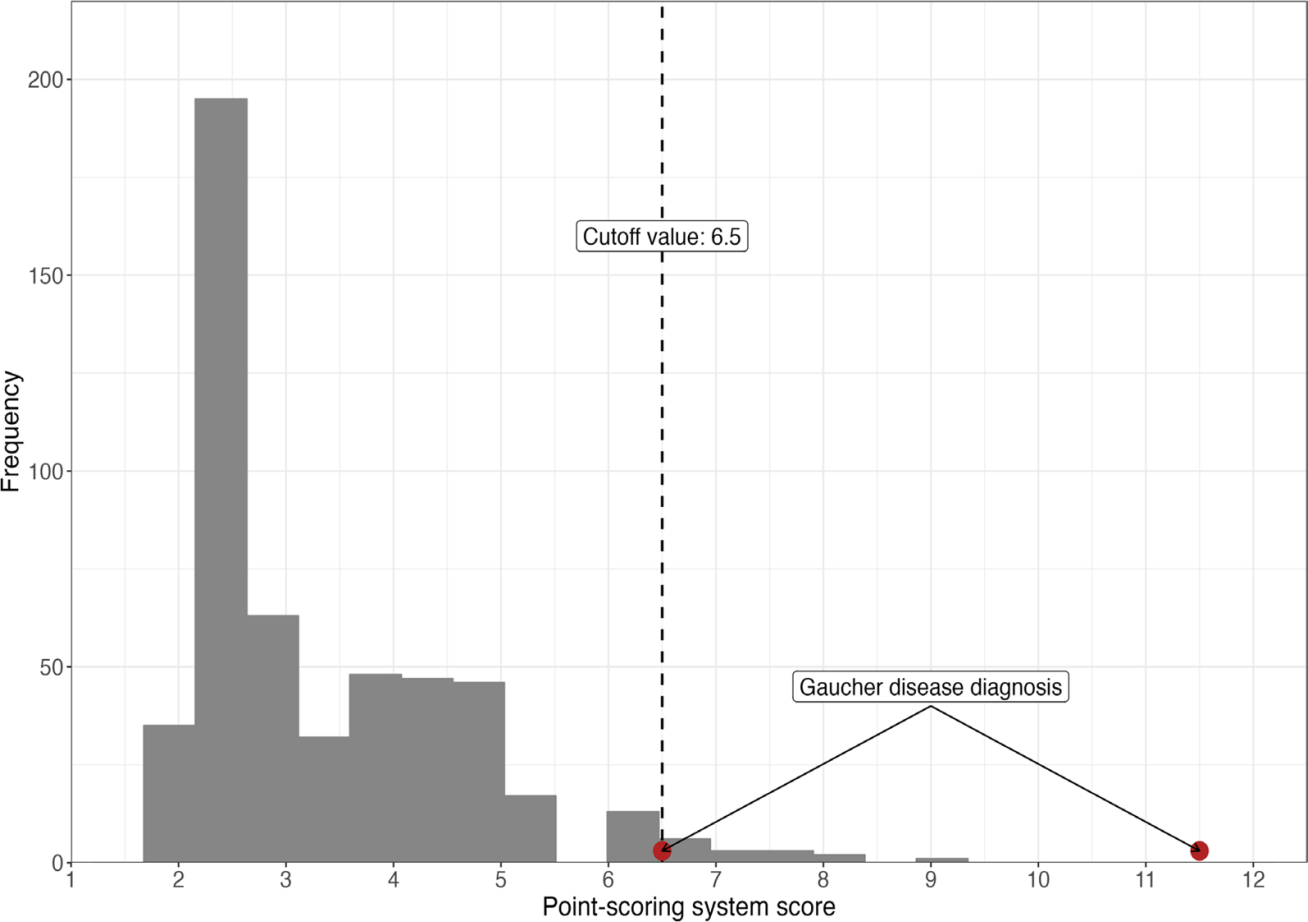
Histogram of proto-type point scoring system

**Fig. 2:**
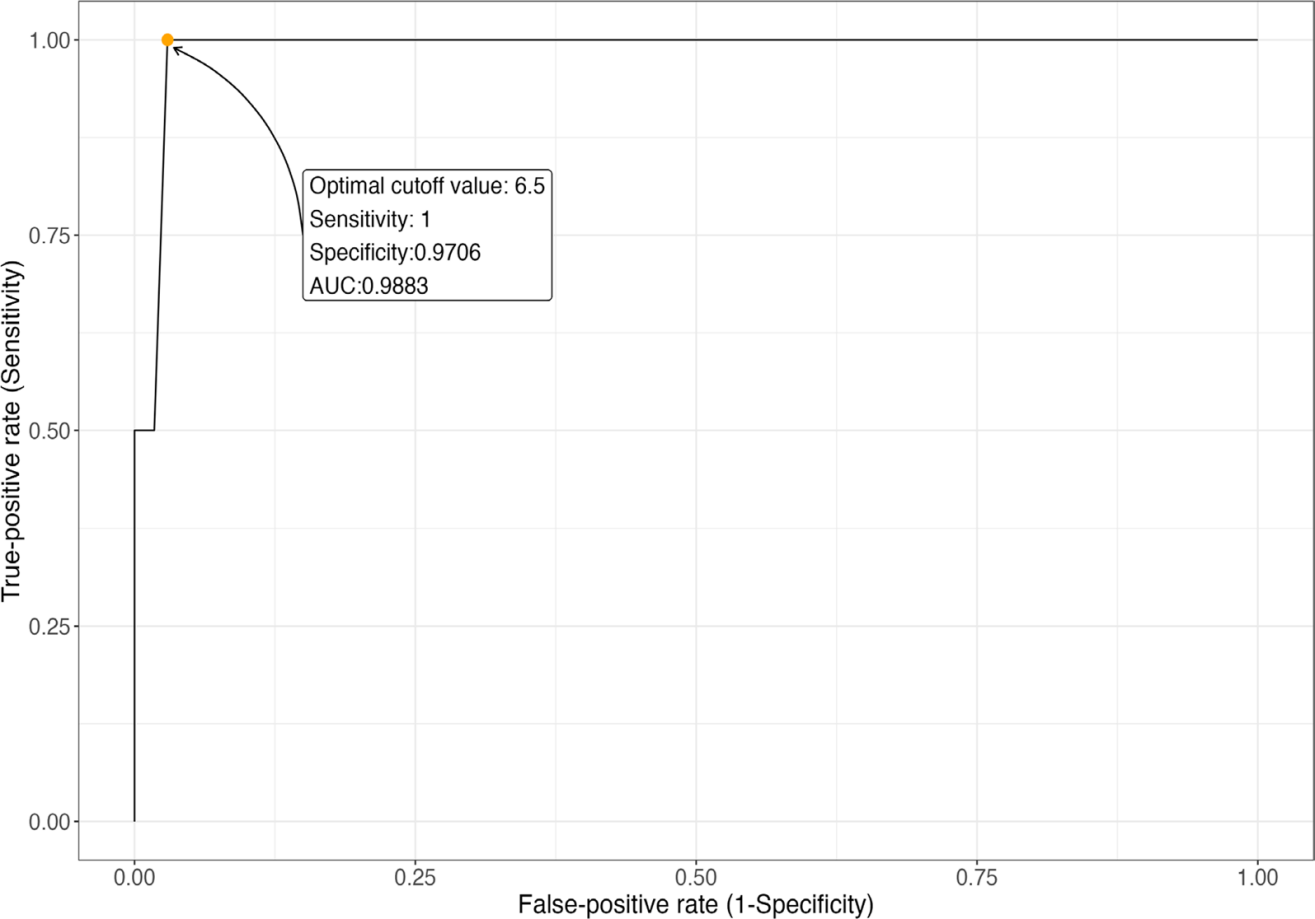
Receiver-operating characteristic (ROC) curve

The ROC curve and cutoff value for the GD PSS score were derived from analyzing 513 participants. A histogram showed that the PSS scores of the two GD patients were distinct from those of other participants (Fig. 1). The cutoff point was identified as 6.5, with an AUC of 0.9883 (95% CI: 0.9677-1). The sensitivity and specificity at this cutoff were 1.0 and 0.9706, respectively (Fig. 2).

### Prospective diagnosis of GD in two patients

Two patients were diagnosed with GD during prospective enrollment. The PSS scores were clearly distinct between GD and non-GD patients, as described in Table 3. Among the two confirmed cases, the first patient was a 23-year-old male with mild thrombocytopenia, disturbed motor function, myoclonic epilepsy, and cognitive deficit, yielding a PSS score of 6.5. The second was a 7-month-old infant with severe splenomegaly, thrombocytopenia, leukopenia, mild hyperferritinemia, hepatomegaly, disturbed motor function, and growth retardation, resulting in a PSS score of 11.5.

**Table 3:** Comparison between non-Gaucher patients with Gaucher disease patients

Characteristic	Non-Gaucher (N = 511)	Gaucher (N = 2)
Cohort		
Prospective	349 (68%)	2 (100%)
Retrospective	162 (32%)	0 (0%)
Overall PSS score		
Mean (SD)	3.47 (1.26)	9.00 (3.54)
Range	2.00, 9.00	6.50, 11.50
Median (IQR)	3.00 (2.50, 4.50)	9.00 (7.75, 10.25)
Assessed GED-C PSS sign or co-variables		
Splenomegaly (≥ 3 × normal)	17 (3.3%)	1 (50%)
Disturbed oculomotor function (slow horizontal saccades with unimpaired vision)	2 (0.4%)	0 (0%)
Thrombocytopenia	281 (55%)	2 (100%)
Anaemia	343 (67%)	0 (0%)
Leukopenia	160 (31%)	1 (50%)
Hyperferritinaemia	65 (13%)	1 (50%)
Hepatomegaly	61 (12%)	1 (50%)
Dyslipidemia	13 (2.5%)	0 (0%)
Elevated angiotensin-converting enzyme levels	2 (0.4%)	0 (0%)
Family history of Gaucher disease	0 (0%)	0 (0%)
Disturbed motor function (impairment of primary motor development)	1 (0.2%)	2 (100%)
Myoclonus epilepsy	13 (2.5%)	1 (50%)
Cognitive deficit	5 (1.0%)	1 (50%)
Gammopathy—monoclonal or polyclonal	3 (0.6%)	0 (0%)
Bone issues, including pain, crises, avascular necrosis, and fractures	6 (1.2%)	0 (0%)
Kyphosis	0 (0%)	0 (0%)
Low bone mineral density	3 (0.6%)	0 (0%)
Jewish ancestry	0 (0%)	0 (0%)
Gallstones	5 (1.0%)	0 (0%)
Bleeding, bruising, or coagulopathy	55 (11%)	0 (0%)
Growth retardation including low body weight	10 (2.0%)	1 (50%)
Asthenia	4 (0.8%)	0 (0%)
Cardiovascular calcification	0 (0%)	0 (0%)
Fatigue	17 (3.3%)	0 (0%)
Pulmonary infiltrates	0 (0%)	0 (0%)
Age ≤ 18 years	448 (88%)	1 (50%)
Family history of Parkinson disease	0 (0%)	0 (0%)
Blood relative who died of fetal hydrops and/or with diagnosis of neonatal sepsis of uncertain etiology	0 (0%)	0 (0%)

GED-C, Gaucher Early Diagnosis Consensus; PSS, point-scoring system

## Discussion

The GED-C PSS was first validated in the UK with 25 patients [13] and in a biobank study of Finland [14]. The UK study found that the mean PSS score (divided by the number of factors included) in GD patients was 1.08 (standard deviation, 0.25) compared to 0.58 (standard deviation, 0.31) in non-GD individuals, while a PSS score of 0.82 distinguished GD with a sensitivity of 100% and a specificity of 71% [13]. The Finland study, utilizing both retrospective data from 170,000 adults in collaboration with the Finland Biobank and prospective data from new patients, discovered an indicative PPS of 6–18.5 for confirmed GD patients [14].

In contrast to the other two earlier studies, this Korean study differed in that most data were collected prospectively based on physicians’ suspicions. The number of GD patients diagnosed was low, with only two confirmed cases. The global incidence of type I GD is 1 in 1,000 in Ashkenazi Jews, generally 1 in 30,000 to 40,000 among other ethnicities, and types 2 and 3 occur even less frequently [1, 2]. Considering the low incidence rate, statistically, it is possible that no patients would have been diagnosed with GD in the study. However, this study demonstrated that when clinically suspected patients are screened, the diagnosis can be made at a much higher rate. This suggests that the real-world implementation of GED-C PPS allows for determining a diagnosing threshold, as demonstrated in previous studies.

One pediatric patient with type 2 GD and one adult with type 3 GD were diagnosed in the study. We observed that the PSS of these two GD patients deviated significantly from that of the non-GD group. As the diverse phenotypes of type 3 GD may hinder a timely diagnosis if not properly suspected, GED-C PSS could be used as a tool to assist diagnosis.

This study was also unique in that most of the participants were pediatric patients, demonstrating the applicability of GED-C PSS in this age group. Globally, while some countries have included Gaucher disease (GD) in their newborn screening programs, the majority have not. In such settings, it is crucial for clinicians to actively suspect and diagnose GD. Therefore, the significance of our study lies in demonstrating that the GED-C PSS can serve as a helpful tool in identifying and diagnosing this rare condition in real-world clinical practice.

This study has several limitations. First, we established age-specific hemoglobin criteria for anemia, distinct from the original GED-C consensus, in order to diagnose anemia in pediatric patients. Second, the potential for variability in the results could have arisen due to differences in the screening items used among the various physicians involved. However, in real-world clinical practice, this approach can be reflective of it. Lastly, including more patients would have resulted in the identification of more cases and thus more reliable results.

## Conclusions

This study with over five hundred patients was the first of its kind to investigate the clinical use of GED-C PSS prospectively, as opposed to earlier studies. Approaches including newborn screening, diagnostic algorithms, and scoring-based screening as used in this study, should be considered for timely GD diagnosis given its prevalence and available resources. This study provides direct evidence that GED-C scoring can be used for diagnosing GD in both children and adults.

## Data Availability

Data is available upon request to the corresponding author.

## Abbreviations

GD: Gaucher Disease
GED-C: Gaucher Earlier Diagnosis Consensus
PSS: Point-Scoring System
NBS: Newborn Screening
IRB: Institutional Review Board
ROC: Receiver Operating Characteristic
AUC: Area Under the Curve
CI: Confidence Interval
SD: Standard Deviation
IQR: Interquartile Range
ACE: Angiotensin-Converting Enzyme
